# The impact of the COVID-19 pandemic in diabetes and dyslipidemia management in a Spanish region: a retrospective study of the Aragon population

**DOI:** 10.3389/fmed.2023.1191026

**Published:** 2023-07-06

**Authors:** Rocio Mateo-Gallego, Irene Gracia-Rubio, María Carmen Garza, Alberto Cebollada, Sofía Pérez-Calahorra, Ana Bayona-Sánchez, Cristina Bujeda-Hernández, Estibaliz Jarauta, Maria Antonia Sánchez-Calavera, Itziar Lamiquiz-Moneo

**Affiliations:** ^1^Hospital Universitario Miguel Servet, Instituto de Investigación Sanitaria Aragón (IIS Aragón), CIBERCV, Zaragoza, Spain; ^2^Department of Physiatry and Nursing, Faculty of Health and Sports Sciences, University de Zaragoza, Huesca, Spain; ^3^Department of Human Anatomy and Histology, School Medicine, University of Zaragoza, Zaragoza, Spain; ^4^Computation Unit, Instituto Aragonés de Ciencias de la Salud (IACS Aragón), Zaragoza, Spain; ^5^Department of Physiatry and Nursing, Faculty of Health Sciences, University of Zaragoza, Zaragoza, Spain; ^6^Department of Medicine and Psychiatry, University of Zaragoza, Zaragoza, Spain; ^7^Health Research Institute of Aragon (IIS Aragón), Zaragoza, Spain; ^8^Research Network on Preventive Activities and Health Promotion (Red de Investigación en Actividades Preventivas y Promoción de la Salud), Barcelona, Spain; ^9^Aragones Health Service, Zaragoza, Spain

**Keywords:** type 2 diabetes, dyslipidemia, COVID-19 pandemic, health system overload, chronic disease

## Abstract

**Introduction:**

Previous research has indicated that the COVID-19 outbreak had a negative impact on the diagnosis and management of cardiometabolic diseases. Our aim was to analyze the impact of the COVID-19 pandemic on the management of dyslipidemia and type 2 diabetes (T2D) in the Aragon region of Spain.

**Methods:**

We conducted an observational retrospective study, which included data from all patients diagnosed with active T2D or dyslipidemia in Aragon during 2019–2021. Data was collected from the BIGAN platform, a big database that includes all healthcare data from the Aragon population. Clinical, biochemical, and pharmacological prescription information was obtained for each patient and for each year.

**Results:**

Out of the total population of 1,330,000 in the Aragon region, 90,000 subjects were diagnosed with T2D each year, resulting in a prevalence of approximately 7%. The COVID-19 pandemic resulted in a decrease in the prevalence of this disease and a lower incidence during the year 2020. In addition, patients with T2D experienced a deterioration of their glucose profile, which led to an increase in the number of patients requiring pharmacological therapy. The prevalence of dyslipidemia was approximately 23.5% in both 2019 and 2020 and increased to 24.5% in 2021. Despite the worsening of the anthropometric profile, the lipid profile improved significantly throughout 2020 and 2021 compared to 2019. Moreover, the number of active pharmacological prescriptions increased significantly in 2021.

**Discussion:**

Our findings suggest that the overload of the health system caused by the COVID-19 pandemic has resulted in an underdiagnosis of T2D. Moreover, patients with T2D experienced a worsening of their glycemic profile, an increase in their pharmacological requirements, and lower performance of their analytical determinations. Dyslipidemic subjects improved their lipid profile although the value of lipid profile determination decreased between 2020 and 2021.

## 1. Introduction

Since its first recognition in Wuhan, China, in December 2019, the COVID-19 pandemic caused by the new severe acute respiratory syndrome-Coronavirus-2 (SARS-CoV2) has rapidly spread worldwide. The first reported case outside China occurred in Thailand in January 2020. Due to the continuous increase in cases, a complete lockdown in Wuhan was declared in January 2020 (1–3). Currently, the pandemic has spread to more than 200 countries, causing 464 million cases and approximately 6 million deaths worldwide (4). Spain is one of the European countries that has been most affected. From the moment the pandemic was declared in August of 2022, Spain had a prevalence of 13.3 million cases, of which 111,000 have died (mortality <1%) (5). Specifically, in Aragon, there were 410,000 confirmed cases and 4,649 deaths, representing 1.15% (6).

In the absence of effective treatments or vaccines, measures such as social distancing and lockdowns of large sections of society have been implemented to slow the spread of the viral infection. In Spain, a nationwide lockdown was imposed in 2020 between March 9th and June 21st. This period caused people to limit their daily routine activities, resulting in changes in eating habits, decreased physical activity, increased weight gain, and difficulty in access to medications (7–9). In addition, health system overload meant that routine controls were no longer performed on individuals with chronic diseases such as type 2 diabetes (T2D) and dyslipidemia. Several studies have reported different results regarding the effect of the lockdown on T2D patients. For example, Falcetta et al. concluded that the lockdown did not exert a negative effect on glycemic control in patients with T2D in Italy (10). In another study, Karatas et al. demonstrated that prolonged lockdown due to the COVID-19 pandemic worsened glucose regulation and increased triglyceride levels in patients with T2D, independent of weight gain (11). Remarkably, this study analyzed the effect of a specific period of lockdown in populations with T2D but not in the mid-term. Regarding dyslipidemia, one study analyzed the effect of COVID-19 on lipid and anthropometric profiles in children with pre-existing dyslipidemia (12). However, there are few studies that analyzed the effect of COVID-19 on a huge older population with previous chronic diseases such as diabetes or dyslipidemia. Therefore, our study aimed to analyze the effects of the COVID-19 pandemic on patients with T2D and/or dyslipidemia in a Spanish region, evaluating the clinical, biochemical, and pharmacological variation of these individuals over the period from 2019 to 2021.

## 2. Materials and methods

### 2.1. Study design

We conducted a retrospective observational study including all subjects with an active diagnosis of T2D and/or dyslipidemia during the years 2019–2021 from Aragon. The Spanish public health system distributes the population into different health districts, with approximately 20,000–30,000 patients per district. They share a single primary care center, a single laboratory with computerized data, and a central drug registry. All these data have recently been combined, allowing clinical, biochemical, and pharmacological data to be obtained from all patients with a health card in the community of Aragon, which has a population of approximately 1,330,000 inhabitants.

This information was obtained from the BIGAN platform, in which demographics, clinical, and analytical values were requested from all users with an Aragon health card from their primary care provider during their annual follow-up visits. BIGAN is the Big Data project of the Department of Health of the Government of Aragon, created to improve health care using the data that is routinely collected within the Aragon public system. BIGAN is a technological infrastructure owned by the Government of Aragon, managed by the Aragon Institute of Health Sciences, and financed by the Department of Health and the Aragon Health Service, in which information from health information systems is obtained. This platform allows for obtaining clinical, biochemical, and pharmacological data in a pseudonymous manner from all the data collected by the Department of Health and the Aragon Health Service (13). The study protocol was approved by the Clinical Research Ethics Committee of Aragon (PI002/22).

We included data from all patients with an active diagnosis of T2D and/or dyslipidemia from the BIGAN platform, who were attending the public health primary care centers in Aragon during the years 2019–2021. Diabetes diagnosis is indicated with the code T90, while dyslipidemia is indicated with the T93 code. We recruited all patients with T90 and or T93 diagnosis codes indicated for each year 2019–2021.

### 2.2. Participants

Patients with T2D were defined according to the standards of the American Diabetes Association (ADA) (12) as those individuals who met any of the following criteria: a fasting blood glucose level greater than 126 mg/dL measured at two separate times or a glycosylated hemoglobin (HbA1c) > 6.5%. We defined patients with poorly controlled diabetes as those whose HbA1c levels were greater than 8%, and those who did not meet the therapeutic goals were defined by an HbA1c level higher than 7%. We obtained analytical variables (including glucose and HbA1clevels), anthropometric variables [body weight, sex, and body mass index (BMI)], and active T2D drugs for each subject. We defined active T2D drugs such as hypoglycemic medication that was prescribed for each T2D patient with a previous start date and completion date after each year studied (2019, 2020, and 2021).

Patients with dyslipidemia were defined according to the Spanish Cardiology Society and Spanish Atherosclerosis Society criteria (13). The clinical guideline defined them as patients who meet the following criteria: (1) subjects with low-density lipoprotein (LDL) cholesterol >116 mg/dL and no cardiovascular risk factors; (2) subjects with T2D with less than ten years of evolution and LDL cholesterol >100 mg/dL; (3) subjects with LDL cholesterol >70 mg/dL with a high risk of cardiovascular disease or long-standing T2D or renal disease or hypertension; (4) individuals with LDL cholesterol >50 mg/dL in secondary prevention or with T2D with target organ damage or severe chronic kidney disease. For those subjects diagnosed with dyslipidemia, we obtained analytical variables (total cholesterol, triglycerides, high-density lipoprotein (HDL) cholesterol, and LDL cholesterol), anthropometric parameters (body weight, sex, and BMI), and active lipid-lowering drugs for each patient. Body weight should be determined following the protocol at each face-to-face visit by the primary care professional. In addition, patients with diabetes and or dyslipidemia must attend a face-to-face visit at least every year. We defined patients with poorly controlled dyslipidemia as those with LDL cholesterol levels greater than the 95th percentile adjusted by age and sex for the Spanish population (14).

### 2.3. Laboratory measurements

Blood samples were collected by venipuncture after overnight fasting. Blood glucose concentration was measured with the glucose-oxidase method. HbA1c levels were determined *via* high-performance liquid chromatography. Triglycerides, total cholesterol, and HDL-cholesterol levels were measured by spectrophotometry (AU5800 Beckman Coulter Inc.). LDL cholesterol levels were calculated using the Friedewald formula.

### 2.4. Medication registry

Both hypoglycemic and lipid-lowering drugs prescribed by the public health system services were recorded by the Aragon Health Service electronic system using an ATC code system. The lipid-lowering drugs included simvastatin, lovastatin, pravastatin, fluvastatin, atorvastatin, rosuvastatin, pitavastatin, and ezetimibe, as well as the combinations of simvastatin with ezetimibe, atorvastatin with ezetimibe, and rosuvastatin with ezetimibe. Hypoglycemic drugs included monotherapy pharmacology, such as biguanide, sulfonylureas, dipeptidyl peptidase-4 inhibitors (IDPP-4), sodium-glucose cotransporter type 2 inhibitors (ISGLT-2), glucagon-like peptide 1 (GLP-1) agonists, glucosidase inhibitors, meglitinide, and thiazolidinedione, as well as dual therapy, such as biguanide and ISGLT-2, IDPP-4, and ISGLT-2, thiazolidinedione and IDPP-4, biguanide and IDPP-4, biguanide and thiazolidinedione, and different analogous of insulin.

### 2.5. Statical analysis

Continuous variables were expressed as mean ± SD or median (25th percentile to 75th percentile), and categorical (nominal) variables were reported as percentages of the total sample. Differences between independent variables were calculated by the T-Student or the Mann–Whitney U test, as appropriate, while categorical variables were compared using the Chi-squared test. Differences between more than two independent variables were calculated using ANOVA or the Kruskal-Wallis test. All statistical analyses were performed using R version 3.5.0 (15), and the significance level was set at *p* < 0.05.

## 3. Results

### 3.1. Diabetic population

In 2019, the population of Aragon had 91,120 individuals with an active diagnosis of T2D, resulting in a prevalence of 6.85%; whereas, in 2020, this decreased to 90,344 subjects, with a prevalence of 6.79%. However, in 2021, there was an increase of 1,447 patients with T2D, reaching a prevalence of 6.99% (*p* < 0.001). Throughout 2020, 4,049 new patients with T2D were diagnosed, with an incidence of 3.26 new cases per 100,000 inhabitants. The incidence increased significantly in 2021, when 5,998 new cases were diagnosed, increasing the incidence to 4.91 cases per 100,000 inhabitants (*p* < 0.001).

In 2019, 6269 patients (6.78%) with T2D did not have their glucose level measured, and this percentage increased significantly to 8.10 and 8.76% in 2020 and 2021, respectively (*p* < 0.001). Regarding Hb1Ac, in 2019, 10,609 subjects (11.64%) did not have any Hb1Ac levels determined, and this percentage increased significantly to 13.74 and 14.48% in 2020 and 2021, respectively (*p* < 0.001).

Patients with T2D were mostly men with a median age below 70 years. They were markedly overweight, with mean glucose concentrations greater than 130 mg/dL and HbA1c higher than 6.7%. More than 10% of the diabetic population had not been prescribed any hypoglycemic medication, whereas 45% had been prescribed monotherapy, 20% were under combined therapies, and 25% were under an analog of insulin treatment (Table 1). In addition, the average age of T2D in men was 67 years, significantly lower than that in women, who have an average age of more than 71 years (*p* < 0.001, Supplementary Figure S1A). In addition, it is interesting to highlight that only 50636 T2D patients (55.5%) had determined their BMI in 2019 although this percentage rose to 66.9% in the years 2020 and 2021, with 60485 and 61478 T2D patients who had their BMI value determined in those years, respectively.

**Table 1:** Clinical, biochemical, and hypoglycemic medications of all subjects with type 2 diabetes in Aragon.^1^

		2019 (*N* = 91,120)	2020 (*N* = 90,344)	2021 (*N* = 91,791)
Age, years		69.3 ± 14.2	69.3 ± 14.3	69.3 ± 14.3
Men, *n* (%)		50,900 (55.8)	50,625 (56.0)	51,475 (56.1)
Body weight, kg		79.1 ± 16.22	79.1 ± 16.2	79.2 ± 16.6
BMI, kg/m^2^		29.4 (26.3–32.9)	29.4 (26.4–32.9)	29.4 (26.3–32.9)
Glucose, mg/dL		131 (111–156)	131 (111–158)	131 (112–157)
Hb1Ac, %		6.70 (6.18–7.51)	6.70 (6.15–7.50)	6.73 (6.18–7.52)
T2D drugs therapy, *n* (%)	Without medication	9,792 (10.7)	9,670 (10.7)	90,326 (10.2)
	Insulin	22,726 (24.9)	22,619 (25)	22,761 (24.8)
	Biguanide	32,770 (35.9)	31,011 (34.3)	31,025 (33.8)
	Sulfonylureas	3,400 (3.73)	2,949 (3.26)	2,555 (2.78)
	IDPP 4	4,628 (5.07)	4,932 (5.46)	5,239 (5.71)
	ISGLT 2	637 (0.69)	859 (0.95)	1,256 (1.37)
	GLP1 agonist	233 (0.25)	317 (0.35)	505 (0.55)
	Glucosidase Inhibitor	97 (0.11)	72 (0.08)	58 (0.06)
	Meglitinide	656 (0.71)	592 (0.65)	495 (0.54)
	Thiazolidinedione	135 (0.14)	131 (0.14)	129 (0.14)
	Biguanide and ISGLT 2	2,460 (3.69)	3,525 (3.90)	4,926 (5.4)
	IDPP4 and ISGLT2	13 (0.01)	18 (0.01)	27 (0.03)
	Thiazolidinedione and IDPP4	71 (0.07)	96 (0.11)	125 (0.14)
	Biguanide and IDPP4	13,272 (14.6)	13,343 (14.7)	13,168 (14.3)
	Biguanide and thiazolidinedione	230 (0.25)	210 (0.23)	196 (0.21)

^1^Quantitative variables are expressed as means ± standard deviations, except for variables not following normal distribution that are expressed as medians (interquartile ranges). Qualitative variables are expressed as percentages. BMI: Body Mass Index; T2D: Type 2 Diabetes; Hb1Ac: Hemoglobin A1c; IDPP 4: Dipeptidyl Peptidase-4 inhibitors; ISGLT 2: Sodium-glucose Cotransporter-2 inhibitors and GLP1: Glucagon-like peptide-1.

Among the patients diagnosed with T2D, 81,933 individuals remained with the disease throughout the analyzed years. These patients were, in 2019, significantly younger, with higher body weight and BMI and higher glucose levels than the total T2D population that had only been actively diagnosed in 2019 (*p* < 0.001, *p* < 0.001, *p* = 0.023, and *p* < 0.001, respectively, Supplementary Table S1).

**Figure 1:**
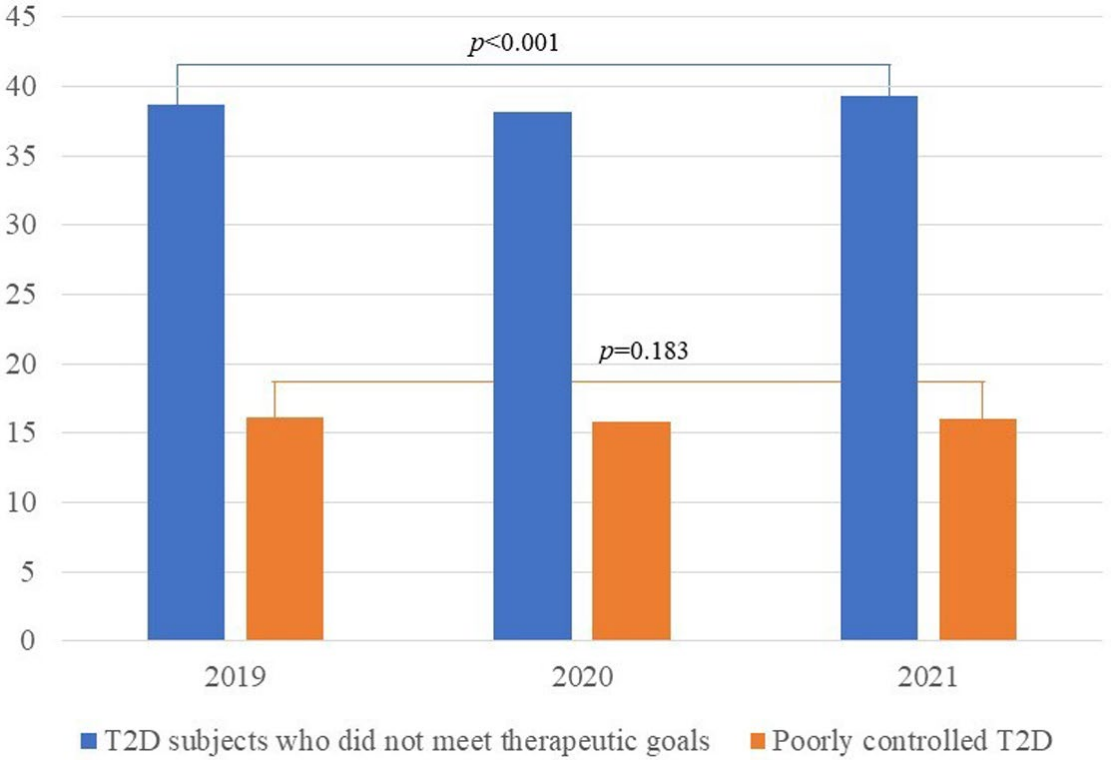
Evolution of control of T2D subjects throughout the follow-up.

All patients with an active T2D diagnosis experienced a significant decrease in body weight as well as BMI, but their glucose and Hb1Ac levels modestly but significantly increased (*p* < 0.001 in all cases, Table 2). Regarding medication, there was a significant decrease in patients with T2D who were able to control the disease through exercise and diet. In addition, the number of patients with T2D who received monotherapy decreased from 47.4% in 2019 to 43.2% in 2021, especially with a reduction in the prescription of biguanide and sulfonylureas. In contrast, the prescription of dual therapy, particularly the prescription of biguanide and ISGLT2 and thiazolidinedione and IDPP-4, significantly increased (*p* < 0.001 for all cases). The number of poorly controlled patients did not vary throughout the years analyzed (*p* = 0.183). However, the number of subjects with T2D who did not meet therapeutic goals varied significantly throughout the years 2019 (38.7%), 2020 (38.2%), and 2021 (39.3%) (p < 0.001, Figure 1).

**Table 2:** Clinical, biochemical, and hypoglycemic medications of all subjects with active type 2 diabetes diagnosis from 2019 to 2021 in Aragon.^1^

		2019 (*N* = 81,933)	2020 (*N* = 81,933)	2021 (*N* = 81,933)	*p*^2^
Age, years		68.1 ± 14.0	69.1 ± 14.0	70.1 ± 14.0	NA
Body weight, kg		79.5 ± 16.1	78.9 ± 15.9	78.5 ± 16.1	<0.001
BMI, kg/cm^2^		29.5 (26.5–33.0)	29.4 (26.4–32.8)	29.2 (26.2–32.6)	<0.001
Glucose, mg/dL		140 ± 46.0	141 ± 46.8	141 ± 47.7	<0.001
Hb1Ac, %		7.00 ± 1.33	6.98 ± 1.26	7.03 ± 1.30	<0.001
T2D drugs therapy, *n* (%)	Without medication	8,658 (10.6)	8,386 (10.2)	7,820 (9.54)	<0.001
	Insulin	19,594 (23.9)	20,404 (24.9)	21,468 (26.2)	<0.001
	Biguanide	30,312 (37.0)	28,071 (34.3)	26,141 (31.9)	<0.001
	Sulfonylureas	3,053 (3.73)	2,753 (3.36)	2,476 (3.02)	<0.001
	IDPP 4	3,929 (4.80)	4,426 (5.40)	4,851 (5.92)	<0.001
	ISGLT 2	610 (0.74)	732 (0.89)	901 (1.10)	<0.001
	GLP1 agonist	222 (0.27)	291 (0.36)	401 (0.49)	<0.001
	Glucosidase Inhibitor	83 (0.10)	65 (0.08)	55 (0.07)	0.051
	Meglitinide	532 (0.65)	524 (0.64)	467 (0.57)	0.083
	Thiazolidinedione	124 (0.15)	123 (0.15)	127 (0.16)	0.966
	Biguanide and ISGLT 2	2,346 (2.86)	3,267 (3.99)	4,305 (5.25)	<0.001
	IDPP4 e ISGLT2	12 (0.01)	17 (0.02)	27 (0.03)	0.044
	Thiazolidinedione and IDPP4	67 (0.08)	92 (0.11)	121 (0.15)	<0.001
	Biguanide and IDPP4	12,175 (14.9)	12,581 (15.4)	12,582 (15.4)	0.005
	Biguanide and thiazolidinedione	216 (0.26)	201 (0.25)	191 (0.23)	0.457

^1^Quantitative variables are expressed as means ± standard deviations, except for variables not following normal distribution that are expressed as medians (interquartile ranges). Qualitative variables are expressed as percentages. BMI, Body Mass Index; T2D, Type 2 Diabetes; Hb1Ac, Hemoglobin A1c; IDPP 4, Dipeptidyl Peptidase-4 inhibitors; ISGLT 2, Sodium-glucose Cotransporter-2 inhibitors; GLP1, Glucagon-like peptide-1. ^2^The *p* value was calculated by ANOVA test, Friedmann test, or Chi-square, as appropriate.

### 3.2. Dyslipidemic population

In 2019, the population with dyslipidemia comprised 310,796 patients, with a prevalence of 23.5%. In 2020, patients with dyslipidemia increased to 314,155 subjects, with a prevalence of 23.6%, while in 2021, subjects with dyslipidemia increased to 324,639 reaching a prevalence of 24.5% (*p* < 0.001). In 2020, 11,189 new patients with dyslipidemia were diagnosed, with an incidence of 11.03 new cases per 100,000 inhabitants. In 2021, the incidence increased significantly to 18.3 cases per 100,000 inhabitants (*p* < 0.001) when 18,320 new cases were confirmed.

In 2019, 51,479 subjects with dyslipidemia (16.6%) did not have their total cholesterol levels measured, and this percentage significantly increased to 20.1 and 21.3% in 2020 and 2021, respectively (*p* < 0.001). Regarding HDL-cholesterol, 59,153 subjects (19.0%) did not have their levels measured in 2019, and this percentage significantly increased to 23.1 and 22.5% in 2020 and 2021, respectively (*p* < 0.001). Regarding triglycerides, 55,706 subjects (17.9%) did not have their levels measured in 2019, and this percentage significantly increased to 21.6 and 22.8% in 2020 and 2021, respectively (*p* < 0.001). Finally, LDL-cholesterol levels were not measured in 82,735 subjects (26.6%) in 2019, and this percentage increased significantly to 32.0 and 37.2% in 2020 and 2021, respectively (*p* < 0.001).

The population with dyslipidemia was predominantly women, with a median age of 63 years and marked overweight although with a lower BMI than the T2D population. In addition, these populations have determined their BMI values at 224,336 (72.2%), 257,220 (81.9%), and 264,059 (81.3%) dyslipidemic subjects in the years 2019, 2020, and 2021, respectively. Their total cholesterol levels were slightly above 200 mg/dL, LDL-cholesterol levels were greater than 123 mg/dL, HDL-cholesterol levels were higher than 55 mg/dL, and their mean triglyceride levels were slightly below 140 mg/dL. Notably, more than 40% of the population had not been prescribed any lipid-lowering medication. The most commonly used drug was atorvastatin, followed by simvastatin and rosuvastatin, whereas the least used drugs were lovastatin and fluvastatin. In addition, the number of poorly controlled patients with dyslipidemia remained constant (> 3% of the population during the analyzed years) (Table 3). Finally, the mean age of men diagnosed with dyslipidemia was approximately 60 years old, significantly lower than that of women whose mean age of diagnosis was greater than 65 years (*p* < 0.001, Supplementary Figure S1B).

**Table 3:** Clinical, biochemical, and lipid-lowering medications of all subjects with dyslipidemia in Aragon.^1^

		2019 (*N* = 310,796)	2020 (*N* = 314,155)	2021 (*N* = 324,639)
Age, years		62.9 ± 15.4	63.2 ± 15.4	63.2 ± 15.4
Men, *n* (%)		151,724 (48.8)	153,088 (48.7)	157.306 (48.5)
Body weight, kg		76.5 ± 15.8	76.7 ± 15.9	77.0 ± 16.4
BMI, kg/cm^2^		28.6 (25.7–32.0)	28.8 (25.8–32.2)	28.7 (25.7–32.1)
Total cholesterol, mg/dL		206 ± 45.4	205 ± 46.2	206 ± 46.5
LDL cholesterol, mg/dL		125 ± 39.2	123 ± 39.7	123 ± 39.9
HDL cholesterol, mg/dL		55.7 ± 18.2	55.4 ± 15.0	55.8 ± 15.2
Triglycerides, mg/dL		139 ± 94.4	139 ± 93.7	138 ± 94.5
Lipid-lowering drugs, *n* (%)	Without medication	133,628 (43.0)	137,857 (43.8)	139,402 (43.9)
	Simvastatin	64,984 (20.9)	60,299 (19.2)	58,886 (19.2)
	Lovastatin	881 (0.28)	756 (0.24)	664 (0.24)
	Pravastatin	5,516 (1.77)	4,884 (1.55)	4,589 (1.55)
	Fluvastatin	1,697 (0.54)	1,489 (0.47)	1,310 (0.47)
	Atorvastatin	66,780 (21.5)	65,169 (20.7)	67,633 (20.7)
	Rosuvastatin	29,462 (9.47)	30,969 (9.86)	35,913 (9.86)
	Pitavastatin	10,179 (3.27)	9,950 (3.17)	10,109 (3.17)
	Ezetimibe	8,313 (2.67)	8,084 (2.57)	8,555 (2.57)
	Simvastatin + Ezetimibe	3,095 (0.996)	2,805 (0.893)	2,740 (0.893)
	Atorvastatin + Ezetimibe	5,853 (1.88)	6,131 (1,95)	6,898 (1.95)
	Rosuvastatin + Ezetimibe	2,925 (0.94)	4,589 (1.46)	8,465 (1.46)
Subjects with LDL cholesterol above the 95th percentile adjusted by age and sex, n (%)		7,398 (3.27)	6,819 (3.21)	6,630 (3.28)

^1^Quantitative variables are expressed as means ± standard deviations, except for variables not following normal distribution that are expressed as medians (interquartile ranges). Qualitative variables are expressed as percentages. BMI: Body Mass Index; LDL: low-density lipoprotein; HDL: high-density lipoprotein.

Among the total population with dyslipidemia, 295,362 subjects remained with the disease in the analyzed years. These patients were significantly younger, with higher body weight and higher concentrations of total cholesterol but lower levels of LDL- and HDL-cholesterol than the total T2D population who only had active diabetic disease in 2019 (*p* < 0.001, *p* = 0.016, *p* < 0.001, *p* < 0.001, and *p* < 0.001, respectively, Supplementary Table S2).

All individuals diagnosed with active dyslipidemia from 2019 to 2021 experienced a significant increase in body weight and BMI although their lipid profile significantly improved by decreasing their total LDL-cholesterol and triglyceride levels and increasing their HDL-cholesterol levels (*p* < 0.001 in all cases, Table 4). Regarding medication, the number of subjects under lipid-lowering treatment increased throughout the follow-up period, mainly with combination therapy, such as atorvastatin with ezetimibe or rosuvastatin with ezetimibe, as well as high-potency statins (*p* < 0.001, in all cases, Table 4). Similarly, we found a significant decrease in the proportion of dyslipidemic subjects with their LDL cholesterol levels above the 95th percentile from 3.33% in 2019 to 2.63% in 2021 (*p* < 0.001, Table 4). Finally, the percentage of subjects with extremely high levels of LDL cholesterol who did not receive lipid-lowering drugs decreased significantly throughout the follow-up from 47.5% in 2019 to 39.7% in 2021 (*p* < 0.001).

**Table 4:** Clinical, biochemical, and lipid-lowering medications of all subjects with an active dyslipidemic diagnosis from 2019 to 2021 in Aragon.^1^

		2019 (*N* = 295,362)	2020 (*N* = 295,362)	2021 (*N* = 295,362)	*p*^2^
Age, years		62.9 ± 15.4	63.9 ± 15.4	64.9 ± 15.4	NA
Body weight, kg		76.5 ± 15.8	76.7 ± 15.9	77 ± 16.4	<0.001
BMI, kg/cm^2^		28.6 (25.7–31.9)	28.8 (25.8–32.2)	28.7 (25.7–32.1)	<0.001
Total cholesterol, mg/dL		207 ± 44.9	204 ± 45.5	203 ± 46.0	<0.001
LDL cholesterol, mg/dL		125 ± 39.0	122 ± 39.2	121 ± 39.2	<0.001
HDL cholesterol, mg/dL		55.0 ± 14.9	55.5 ± 14.9	55.7 ± 15.1	<0.001
Triglycerides, mg/dL		139 ± 94.8	138 ± 92.2	137 ± 90.0	<0.001
Lipid-lowering drugs, *n* (%)	Active medication	167,872 (56.8)	166,953 (56.5)	171,854 (58.2)	<0.001
	Simvastatin	61,815 (20.9)	57,312 (19.4)	54,915 (18.6)	<0.001
	Lovastatin	791 (2.68)	707 (2.34)	649 (2.20)	<0.001
	Pravastatin	5,079 (17.2)	4,614 (15.6)	4,392 (14.9)	<0.001
	Fluvastatin	1,562 (5.29)	1,423 (4.82)	1,291 (4.38)	<0.001
	Atorvastatin	62,923 (21.3)	61,422 (20.8)	62,661 (21.2)	<0.001
	Rosuvastatin	28,319 (9.59)	29,354 (9.94)	32,631 (11.0)	<0.001
	Pitavastatin	9,717 (3.29)	9,495 (3.21)	9,479 (3.21)	0.148
	Ezetimibe	7,959 (2.70)	7,773 (2.63)	8,146 (2.76)	0.011
	Simvastatin + Ezetimibe	2,927 (1.00)	2,702 (0.91)	2,644 (0.90)	<0.001
	Atorvastatin + Ezetimibe	5,537 (1.87)	5,839 (1.98)	6,598 (2.23)	<0.001
	Rosuvastatin + Ezetimibe	2,808 (0.95)	4,322 (1.46)	7,644 (2.59)	<0.001
Subjects with LDL cholesterol above the 95th percentile adjusted by age and sex, *n* (%)		7,167 (3.33)	5,830 (2.94)	4,756 (2.63)	<0.001

^1^Quantitative variables are expressed as means ± standard deviations, except for variables not following normal distribution that are expressed as medians (interquartile ranges). Qualitative variables are expressed as percentages. BMI: Body Mass Index; LDL: low-density lipoprotein; HDL: high-density lipoprotein. ^2^The *p* value was calculated by ANOVA test, Friedmann test, or Chi-square, as appropriate.

## 4. Discussion

This is the first study to analyze the impact of the health system overload and lifestyle habits changes caused by the COVID-19 pandemic on the management of chronic diseases, such as T2D and dyslipidemia, in an entire region of Spain. The results clearly show a situation of underdiagnosis of T2D in the year 2020. This scenario of underdiagnosis has also been seen in other pathologies, such as cardiovascular disease (16, 17) or buccal disorders (18), among others. In addition, in 2020 and 2021, subjects with T2D and dyslipidemia worsened their glucose and HbA1c levels and improved their lipid profiles, respectively, when compared to 2019. These patients also underwent fewer biochemical controls than both populations in 2020 and 2021, indicating a worse follow-up of both chronic diseases. This could be directly related to the saturation of the health system and changes in lifestyle habits and behaviors caused by the COVID-19 pandemic.

First, we observed that the T2D population in Aragon decreased by 778 patients between 2019 and 2020, which reduced the prevalence of this disease from 6.85 to 6.79%. These losses could be partially explained as a result of the high number of deaths from COVID-19 in 2020, which accounted for 973 patients in our community from the beginning of the pandemic until June 30, 2020 (19), and the underdiagnosis produced by the overload of the health system (20). However, between 2020 and 2021, there was an increase of 1,447 individuals in the total number of subjects with T2D, increasing the prevalence of this disease from 6.79% in 2020 to 6.99% in 2021. The same trend could be observed in the dyslipidemic population where the prevalence of this disease remained constant (approximately 23.5%) in 2019 and 2020 and significantly increased in 2021 to 24.5%. In the same way as T2D, this change could be explained by the underdiagnosis that occurred during the year 2020 due to the first wave of COVID-19, as has been seen in other pathologies (18, 21).

According to the ADA guidelines, the goal of diabetes treatment should be to maintain an Hb1AC < 7% in those patients diagnosed within the previous 10 years and those with a long life expectancy. In contrast, the therapeutic goal should be established as Hb1AC < 8% in patients with a short life expectancy, disease progression >10 years, or associated complications (12, 20). In this study, patients with T2D showed a worsening of their glucose and Hb1Ac levels. Additionally, there was an increase in the percentage of subjects who did not meet therapeutic goals. The investigations of the COVID-19 pandemic’s effect on T2D management have previously revealed heterogeneous results; some researchers have described a worsening (11, 22–24) while others have reported an improvement (10, 25) although these studies were limited exclusively to the period of confinement. The worsening of the glycemic profile in the diabetic population could be explained by a deterioration of lifestyle habits, including unhealthy dietary patterns and a decrease in physical activity (26). During the pandemic and especially during lockdown, a high percentage of the population reduced their physical activity to a totally sedentary lifestyle (27). However, the lower control and registration of the dietary habits of this population is a reality previously described (20) where only one-third of the 587 diabetics studied in 2018 had a dietary record in 2020.

Regarding the lipid profile, it is noteworthy that the dyslipidemic population reported a significant mean improvement in their lipid profile, which could be explained, in part, because the determinations of these values decreased during 2020 and 2021. In fact, a clear consequence of the overload on the health system was the increase in the number of patients who did not have their total and HDL-cholesterol or triglyceride levels determined, which was approximately 16% in 2019 compared to 22% in 2021. This increase in the number of patients without measurements of their biochemical markers could be caused by the COVID-19 pandemic, which meant 12.7% fewer discharges in 2020 than in 2019. In addition, the greatest decrease in the number of discharges occurred in March, April, and May of 2020 when the first wave of the COVID-19 pandemic occurred (28).

Regarding variations in the anthropometric characteristics, in the case of the dyslipidemic population, the results reported a significant worsening of the anthropometric parameters and an increase in body weight and BMI, which is in line with previous findings (11, 20, 27). However, in the diabetic population, a marked improvement in their anthropometric characteristics was observed, which seems contradictory to the results reported in other studies (11). These discrepancies could partly be explained by the fact that the evaluated period in our study is not restricted to the confinement period but also includes the years 2020 and 2021.

Regarding the evolution of the pharmacological prescription for the diabetic population, it is remarkable that the number of patients without active hypoglycemic treatment significantly decreased throughout the follow-up period. This could be a direct consequence of the lack of control over this population in the year 2020, as indicated above (20), as well as an increase in physical inactivity (27). Similarly, our results showed a significant decrease in monotherapy drugs and an increase in combined drugs, which seems to indicate a greater progression of the disease (29). It is also remarkable that the percentage of patients treated with insulin, which is considered the last pharmacological step, remained stable for the analyzed 3 years (30). Finally, there has been a decrease in the percentage of the population receiving treatments that are considered obsolete, such as sulfonylureas, in favor of an increase in the latest antidiabetic drugs, which are more effective and produce fewer side effects, such as GLP-1 agonists, IDPP-4, or ISGLT-2 (29).

This study has several limitations. First, data was exclusively provided by the BIGAN platform, which in turn depends on primary care professionals properly registering the data of the patients they attended to. This is pivotal for determining BMI or body weight, which is only registered around 55–65% in the T2D population and around 72–82% in the dyslipidemic population and it is included by the primary care physician. In both populations, good control of body weight is essential for the management of both chronic diseases. Therefore, recording the weight at each face-to-face visit must be improved by the primary care physician. However, for analytical values, the results were automatically extracted from the server, so it does not depend on them being registered by the primary care physician and there cannot be errors in the registration. Second, the pharmacological data reported in this study were based on physician prescriptions. However, it cannot be ensured that patients who were prescribed a certain drug are currently and regularly taking it. Finally, it has not been possible to analyze comorbidities associated with T2D or dyslipidemia, which would have allowed us to better define poorly controlled patients.

The COVID-19 pandemic has led to an overload on the health system and changes in lifestyle patterns that seem to negatively affect other chronic diseases diagnosis and management, such as T2D and dyslipidemia. The results of the present study showed an underdiagnosis of T2D in 2020 by generating lower prevalence and incidence of this disease throughout the year. In addition, subjects with T2D had modestly but significantly worsening glucose and Hb1Ac levels, and an increase in the number of individuals who did not achieve therapeutic goals was observed. On the contrary, the dyslipidemic population experienced an improvement in their lipid profile that could be mainly influenced by the large increase in individuals who did not have any lipid determinations during the years 2020 and 2021.

## Data availability statement

The original contributions presented in the study are included in the article/Supplementary materials, further inquiries can be directed to the corresponding authors.

## Ethics statement

The studies involving human participants were reviewed and approved by the Clinical Research Ethics Committee of Aragon approved the study protocol (PI002/22). Written informed consent for participation was not required for this study in accordance with the national legislation and the institutional requirements.

## Author contributions

IL-M: conceptualization, validation, supervision, and project administration. IL-M, RM-G, and IG-R: methodology and writing – original draft preparation. AB-S, CB-H, and MG: software and data curation. AC and IL-M: formal analysis. MS-C and RM-G: investigation. RM-G and IL-M: resources and funding acquisition. MS-C, EJ, and MG: writing – review and editing. MS-C and EJ: visualization. All authors contributed to the article and approved the submitted version.

## Funding

This work was funded by grants from the Gobierno de Aragon, B14-7R, Spain, and the Spanish Ministry of Economy and Competitiveness CIBERCV. These projects are co-financed by Instituto de Salud Carlos III and the European Regional Development Fund (ERDF) of the European Union’s “A Way to Make Europe.” CIBERCV is a project of Instituto de Salud Carlos III.

## Conflict of interest

The authors declare that the research was conducted in the absence of any commercial or financial relationships that could be construed as a potential conflict of interest.

## Publisher’s note

All claims expressed in this article are solely those of the authors and do not necessarily represent those of their affiliated organizations, or those of the publisher, the editors and the reviewers. Any product that may be evaluated in this article, or claim that may be made by its manufacturer, is not guaranteed or endorsed by the publisher.

## References

[1] Chams N Chams S Badran R Shams A Araji A Raad M. COVID-19: a multidisciplinary review. Front Public Health. (2020) 8:383. doi: 10.3389/fpubh.2020.00383, PMID: 32850602 PMC7403483

[2] Shereen MA Khan S Kazmi A Bashir N Siddique R. COVID-19 infection: origin, transmission, and characteristics of human coronaviruses. J Adv Res. (2020) 24:91–8. doi: 10.1016/j.jare.2020.03.005, PMID: 32257431 PMC7113610

[3] Hu B Guo H Zhou P Shi Z-L. Characteristics of SARS-CoV-2 and COVID-19. Nat Rev Microbiol. (2021) 19:141–54. doi: 10.1038/s41579-020-00459-7, PMID: 33024307 PMC7537588

[4] WHO. (2022). 14.9 million excess deaths associated with the COVID-19 pandemic in 2020 and 2021. Available at: https://www.who.int/news/item/05-05-2022-14.9-million-excess-deaths-were-associated-with-the-covid-19-pandemic-in-2020-and-2021 (Accessed August 31, 2022)

[5] COVID-19 Data Explorer. (2023). Our world in data. Available at: https://ourworldindata.org/coronavirus-data-explorer (Accessed August 9, 2022)

[6] Aguilar-Palacio I Maldonado L Malo S Sánchez-Recio R Marcos-Campos I Magallón-Botaya R. COVID-19 inequalities: individual and area socioeconomic factors (Aragón, Spain). Int J Environ Res Public Health. (2021) 18:6607. doi: 10.3390/ijerph18126607, PMID: 34205348 PMC8296401

[7] Ghosal S Arora B Dutta K Ghosh A Sinha B Misra A. Increase in the risk of type 2 diabetes during lockdown for the COVID19 pandemic in India: a cohort analysis. Diabetes Metab Syndr. (2020) 14:949–52. doi: 10.1016/j.dsx.2020.06.020, PMID: 32604013 PMC7303633

[8] Ghosh A Arora B Gupta R Anoop S Misra A. Effects of nationwide lockdown during COVID-19 epidemic on lifestyle and other medical issues of patients with type 2 diabetes in North India. Diabetes Metab Syndr. (2020) 14:917–20. doi: 10.1016/j.dsx.2020.05.044, PMID: 32574982 PMC7265851

[9] Singhai K Swami MK Nebhinani N Rastogi A Jude E. Psychological adaptive difficulties and their management during COVID-19 pandemic in people with diabetes mellitus. Diabetes Metab Syndr. (2020) 14:1603–5. doi: 10.1016/j.dsx.2020.08.025, PMID: 32862099 PMC7443210

[10] Falcetta P Aragona M Ciccarone A Bertolotto A Campi F Coppelli A. Impact of COVID-19 lockdown on glucose control of elderly people with type 2 diabetes in Italy. Diabetes Res Clin Pract. (2021) 174:108750. doi: 10.1016/j.diabres.2021.108750, PMID: 33722703 PMC9754212

[11] Karatas S Yesim T Beysel S. Impact of lockdown COVID-19 on metabolic control in type 2 diabetes mellitus and healthy people. Prim Care Diabetes. (2021) 15:424–7. doi: 10.1016/j.pcd.2021.01.003, PMID: 33441263 PMC7834877

[12] American Diabetes Association Professional Practice Committee. 2. Classification and diagnosis of diabetes: standards of medical care in diabetes-2022. Diabetes Care. (2022) 45:S17–38. doi: 10.2337/dc22-S002 34964875

[13] semFYC - Medicina familiar y comunitaria. (2023). Medicina resolutiva. semFYC. Available at: https://www.semfyc.es (Accessed August 8, 2022)

[14] Gómez-Gerique JA Gutiérrez-Fuentes JA Montoya MT Porres A Rueda A Avellaneda A. Lipid profile of the Spanish population: the DRECE (diet and risk of cardiovascular disease in Spain) study. DRECE study group. Med Clin. (1999) 113:730–5. 10680124

[15] Team RC. R: A language and environment for statistical computing. Vienna, Austria: R Foundation for Statistical Computing (2018).

[16] Ball S Banerjee A Berry C Boyle JR Bray B Bradlow W. Monitoring indirect impact of COVID-19 pandemic on services for cardiovascular diseases in the UK. Heart. (2020) 106:1890–7. doi: 10.1136/heartjnl-2020-317870, PMID: 33020224 PMC7536637

[17] Chagué F Boulin M Eicher J-C Bichat F Saint-Jalmes M Cransac A. Smoking in patients with chronic cardiovascular disease during COVID-19 lockdown. Front Cardiovasc Med. (2022) 9:845439. doi: 10.3389/fcvm.2022.845439, PMID: 35557527 PMC9086588

[18] Emodi-Perlman A Eli I. One year into the COVID-19 pandemic - temporomandibular disorders and bruxism: what we have learned and what we can do to improve our manner of treatment. Dent Med Probl. (2021) 58:215–8. doi: 10.17219/dmp/132896 33974750

[19] Magallón-Botaya R Oliván-Blázquez B Ramírez-Cervantes KL Méndez-López-de-la-Manzanara F Aguilar-Palacio I Casajuana-Closas M. Geographic factors associated with poorer outcomes in patients diagnosed with COVID-19 in primary health care. Int J Environ Res Public Health. (2021) 18:3842. doi: 10.3390/ijerph18073842 33917578 PMC8038835

[20] Cuevas Fernández FJ Gutiérrez Galeote JC García Marrero MR Iglesias Girón MJ Cabrera de León A Aguirre-Jaime A. Impact of the alteration of the continuity of care in diabetes type 2 patients during the COVID-19 pandemic. SEMERGEN. (2022) 48:308–15. doi: 10.1016/j.semerg.2022.02.007 35537930 PMC8938175

[21] Mazzucchi E Torricelli FCM Vicentini FC Marchini GS Danilovic A Batagello CA. The impact of COVID-19 in medical practice. A review focused on urology. Int Braz J Urol. (2021) 47:251–62. doi: 10.1590/S1677-5538.IBJU.2020.99.08, PMID: 32840335 PMC7857770

[22] Khare J Jindal S. Observational study on effect of lock down due to COVID 19 on HBA1c levels in patients with diabetes: experience from Central India. Prim Care Diabetes. (2021) 16:775–9. doi: 10.1016/j.pcd.2020.12.003, PMID: 33419712 PMC7837016

[23] Psoma O Papachristoforou E Kountouri A Balampanis K Stergiou A Lambadiari V. Effect of COVID-19-associated lockdown on the metabolic control of patients with type 2 diabetes. J Diabetes Complicat. (2020) 34:107756. doi: 10.1016/j.jdiacomp.2020.107756, PMID: 33059982 PMC7540191

[24] Ghesquière L Garabedian C Drumez E Lemaître M Cazaubiel M Bengler C. Effects of COVID-19 pandemic lockdown on gestational diabetes mellitus: a retrospective study. Diabetes Metab. (2021) 47:101201. doi: 10.1016/j.diabet.2020.09.008, PMID: 33069845 PMC7557293

[25] Aso Y Iijima T Tomaru T Jojima T Usui I. No negative impact of a National State of emergency by COVID-19 outbreak on hemoglobin A1c levels in patients with type 2 diabetes living in semi-rural Japan. Am J Med Sci. (2021) 362:104–5. doi: 10.1016/j.amjms.2021.03.010, PMID: 33798459 PMC8007532

[26] Guthold R Stevens GA Riley LM Bull FC. Worldwide trends in insufficient physical activity from 2001 to 2016: a pooled analysis of 358 population-based surveys with 1·9 million participants. Lancet Glob Health. (2018) 6:e1077–86. doi: 10.1016/S2214-109X(18)30357-7, PMID: 30193830

[27] Martinez-Ferran M de la Guía-Galipienso F Sanchis-Gomar F Pareja-Galeano H. Metabolic impacts of confinement during the COVID-19 pandemic due to modified diet and physical activity habits. Nutrients. (2020) 12:E1549. doi: 10.3390/nu12061549, PMID: 32466598 PMC7352228

[28] Instituto Nacional de Estadistica. Encuesta de Morbilidad Hospitalaria. Madrid: Instituto Nacional de Estadística (2020).

[29] Quattrocchi E Goldberg T Marzella N. Management of type 2 diabetes: consensus of diabetes organizations. Drugs Context. (2020) 9:212607–25. doi: 10.7573/dic.212607, PMID: 32158490 PMC7048113

[30] Aschner P. New IDF clinical practice recommendations for managing type 2 diabetes in primary care. Diabetes Res Clin Pract. (2017) 132:169–70. doi: 10.1016/j.diabres.2017.09.002 28962686

