# Supplementary material for: The impact of the COVID-19 pandemic in diabetes and dyslipidemia management in a Spanish region: a retrospective study of the Aragon population
Source: Front Med (Lausanne). 2023 Jul 6;10:1191026. doi: 10.3389/fmed.2023.1191026 (PMC10359133; doi:10.3389/fmed.2023.1191026)
Supplement: Supplementary file 1 [file Table_1.DOCX]

**Supplemental Table 1**. Comparation of clinical, biochemical and hypoglycemic medications characteristics at baseline of all subjects with type 2 diabetes diagnosis in 2019 and those that remained with the diagnosis during all follow-up.^1^

|  | | Subjects with diagnosis of T2D in 2019  (N=91120) | Subjects with diagnosis of T2D during follow-up (N=81933) | *p^2^* |
| --- | --- | --- | --- | --- |
| Age, years | | 69.3 ± 14.2 | 68.1 ± 14.0 | <0.001 |
| Men, n (%) | | 50900 (55.8) | 45991 (56.1) | 0.255 |
| Body weight, kg | | 79.1 ± 16.22 | 79.5 ± 16.1 | <0.001 |
| BMI, kg/m^2^ | | 29.4 (26.3-32.9) | 29.5 (26.5 – 33.0) | 0.023 |
| Glucose, mg/dL | | 131 (111-156) | 131 (112-156) | <0.001 |
| Hb1Ac, % | | 6.70 (6.18 -7.51) | 6.70 (6.20 – 7.50) | 0.244 |
| Insulin, IU/mL | | 11.6 (6.74 - 21.0) | 11.7 (6.78 – 21.2) | 0.967 |
| T2D drugs therapy,  n (%) | Without medication | 9792 (10.7) | 8658 (10.6) | 0.228 |
|  | Insulin | 22726 (24.9) | 19594 (23.9) | <0.001 |
|  | Biguanide | 32770 (35.9) | 30312 (37.0) | <0.001 |
|  | Sulfonylureas | 3400 (3.73) | 3053 (3.73) | 0.955 |
|  | IDPP 4 | 4628 (5.07) | 3929 (4.80) | 0.007 |
|  | ISGLT 2 | 637 (0.69) | 610 (0.74) | 0.265 |
|  | GLP1 agonist | 233 (0.25) | 222 (0.27) | 0.536 |
|  | Glucosidase Inhibitor | 97 (0.11) | 83 (0.10) | 0.739 |
|  | Meglitinide | 656 (0.71) | 532 (0.65) | 0.076 |
|  | Thiazolidinedione | 135 (0.14) | 124 (0.15) | 0.864 |
|  | Biguanide and ISGLT 2 | 2460 (3.69) | 2346 (2.86) | 0.038 |
|  | IDPP4 e ISGLT2 | 13 (0.01) | 12 (0.01) | 0.947 |
|  | Thiazolidinedione and IDPP4 | 71 (0.07) | 67 (0.08) | 0.777 |
|  | Biguanide and IDPP4 | 13272 (14.6) | 12175 (14.9) | 0.084 |
|  | Biguanide and thiazolidinedione | 230 (0.25) | 216 (0.26) | 0.646 |

^1^Quantitative variables are expressed as means ± standard deviations, except for variables not following normal distribution that are expressed as medians (interquartile ranges). Qualitative variables are expressed as percentage. BMI: Body Mass Index; T2D: Type 2 Diabetes; Hb1Ac: Hemoglobin A1c; IDPP 4: Dipeptidyl Peptidase-4 inhibitors; ISGLT 2: Sodium-glucose Cotransporter-2 inhibitors and GLP1: Glucagon-like peptide-1.

***^2^*** The *p* value was calculated by T-test or U Mann Whitney test or Chi-squared as appropriate.

**Supplemental Table 2**. Comparation of clinical, biochemical and lipid-lowering medications at baseline of all subjects with dyslipidemic diagnosis in 2019 versus dyslipidemic and those that remained with the diagnosis during all follow-up.^1^

|  | | Subjects with diagnosis of dyslipidemia in 2019 (N=310796) | Subjects with diagnosis of dyslipidemia during follow-up (N=295362) | *p^2^* |
| --- | --- | --- | --- | --- |
| Age, years | | 62.9 ± 15.4 | 62.1 ± 15.1 | <0.001 |
| Men, n (%) | | 151724 (48.8) | 144322 (48.9) | 0.727 |
| Body weight, kg | | 76.5 ± 15.8 | 76.7 ± 15.8 | 0.016 |
| BMI, kg/m^2^ | | 28.6 (25.7- 31.9) | 28.7 (25.7-32.0) | 0.297 |
| Total cholesterol, mg/dL | | 206 ± 45.4 | 207 ± 44.9 | <0.001 |
| LDL cholesterol, mg/dL | | 125 ± 39.2 | 125 ± 39.0 | <0.001 |
| HDL cholesterol, mg/dL | | 55.7 ± 18.2 | 55.0 ± 14.9 | <0.001 |
| Triglycerides, mg/dL | | 139 ± 94.4 | 139 ± 94.8 | 0.605 |
| Lipid lowering drugs, n (%) | Without medication | 133628 (43.0) | 127490 (43.2) | 0.185 |
|  | Simvastatin | 64984 (20.9) | 61815 (20.9) | 0.851 |
|  | Lovastatin | 881 (0.28) | 791 (2.68) | 0.245 |
|  | Pravastatin | 5516 (1.77) | 5079 (17.2) | 0.101 |
|  | Fluvastatin | 1697 (0.54) | 1562 (5.29) | 0.361 |
|  | Atorvastatin | 66780 (21.5) | 62923 (21.3) | 0.082 |
|  | Rosuvastatin | 29462 (9.47) | 28319 (9.59) | 0.151 |
|  | Pitavastatin | 10179 (3.27) | 9717 (3.29) | 0.748 |
|  | Ezetimibe | 8313 (2.67) | 7959 (2.70) | 0.631 |
|  | Simvastatin + Ezetimibe | 3095 (0.996) | 2927 (1.00) | 0.849 |
|  | Atorvastatin + Ezetimibe | 5853 (1.88) | 5537 (1.87) | 0.806 |
|  | Rosuvastatin + Ezetimibe | 2925 (0.94) | 2808 (0.95) | 0.700 |

^1^Quantitative variables are expressed as means ± standard deviations, except for variables not following normal distribution that are expressed as medians (interquartile ranges). Qualitative variables are expressed as percentage. BMI: Body Mass Index; LDL: low-density lipoprotein; HDL: High-density lipoprotein.

***^2^*** The *p* value was calculated by T-test or U Mann Whitney test or Chi-squared as appropriate.
