# Supplementary figures and images for: The impact of the COVID-19 pandemic in diabetes and dyslipidemia management in a Spanish region: a retrospective study of the Aragon population
Source: Front Med (Lausanne). 2023 Jul 6;10:1191026. doi: 10.3389/fmed.2023.1191026 (PMC10359133; doi:10.3389/fmed.2023.1191026)

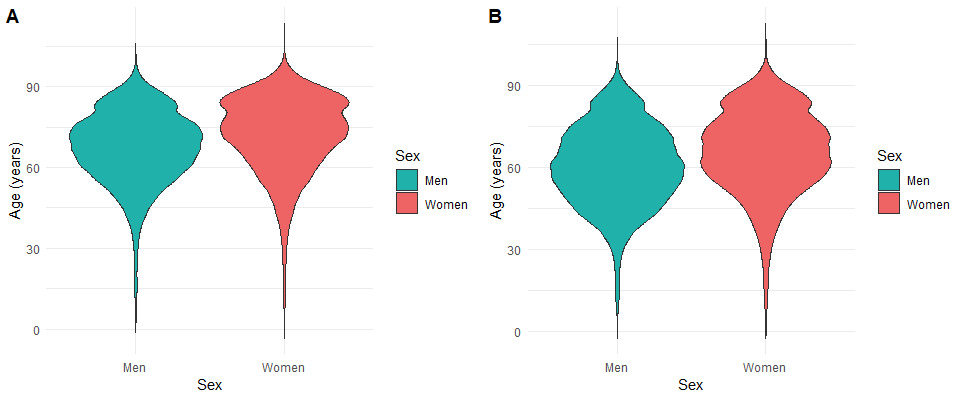

Supplement: Supplementary file 2 [file Image_1.JPEG]

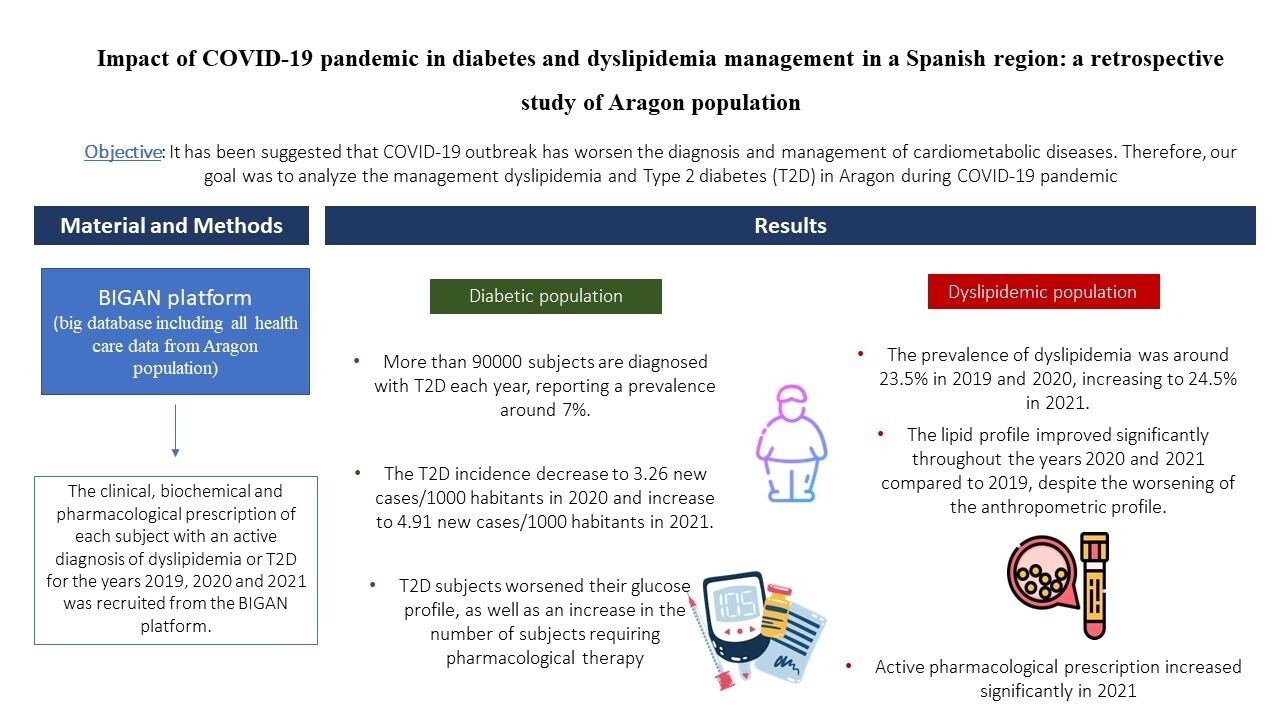

Supplement: Supplementary file 3 [file Image_2.JPEG]
